# The Doctor as a Drug Habitue

**Published:** 1907-08

**Authors:** C. C. Stockard

**Affiliations:** Atlanta, Ga.


### THE DOCTOR AS A DRUG HABITUE.*

BY DR. C. C. STOCKARD, Atlanta, Ga.

I have chosen this subject for a paper with the idea of impressing upon the profession the dangers besetting us in this direction, as shown by the large number of physicians who run into the snares of morphine and other drugs, and, if possible, do something to help check this growing evil, which is already appalling, when we think of the lives probably being sacrificed as a consequence of the work of those whose brains are unreliable in their actions from the effect of drugs.

In order to get at something of an estimate, I wrote to a number of institutions where drug habits are treated, asking the proportion of doctors to the entire number of patients received, and I give below extracts from the replies:—

Dr. Crothers of Hartford says.—

"The statistics you enquire about are difficult to secure without taking a good deal of time and study. I have been in this business thirty-one years, and during that time have averaged from one to two hundred patients a year. Formerly a small percentage of doctors came for treatment, latterly the number has increased, until now about one in every five patients admitted are physicians, some years this is greater than others. In 1905 thirty-one physicians were under treatment, in 1906 twenty-seven. Of course, my statistics only refer to a particular class, and could not be used as representing the general profession.

A friend of mine with a larger experience thinks that ten percent of all drugs habitues are physicians. This may be possibly somewhere near the facts. I should say with a long experience, that fully ten percent, and possibly fifteen use drugs, and at least twenty percent of the entire profession use spirits, either continually or at intervals.

*Read before the Fulton County Medical Society June 20th 1907.

Dr. Pressey of Cleveland.—

Your inquiry in regard to Doctors as drug hasitues is at hand. In reply will say that just about one-half of my drug patients are doctors. However, I would not like you to understand that I mean to convey the impression that one-half the people who take morphine or cocaine are doctors; such a statement would be anything but fair to the profession. You understand that I advertise only in medical journals, these ads seldom come to the eyes of the public. Probably there are twenty-five times as many doctors see my ads as there are of all others. Then there is another reason for the large per cent. of doctors. Other people are as apt or even more so to go to some other place where they will make them more favorable promises than I can make them, while doctors will be more apt to select an ethical place.

Dr. McFarland of Green's Farm, Conn.

Fo rthe past year the doctors have been one in four, or 25 per cent.

Dr. Gundry of Catonsville, Md.

The number of drug cases treated from June 1891 to the present date is 174, 125 men and 49 women. Of the men, 68 were doctors, veterinary surgeon, 3 dentists, one preacher, and 52 laymen, 2 of the latter being sons of physicians, under age and dependent upon their fathers. Of the 49 women 14 were doctors' wives.

Dr. McMillen of Shepard, O.

In reply to your enquiry I should say that about 20 percent of my drug habit cases are physicians.

Dr. Broughton of Rockford, Ill.

As to the percentage of physicians treated here for drug habits on a general average 89 out of 100 are male:

41 of these were for liquor,

48 for opium and other drugs

Of the 48 male drug patients 20 were physicians, which is

20 percent of all patients treated

42 percent of all male drug patients, and

36 percent of all patients treated for drug habits.

This count is taken from 100 consecutive cases and would be a fair average, I believe, for one year's work.

Dr. Williams of the Cincinnati Sanitarium.

Your letter of recent date at hand, and replying to same will say that out of nineteen such cases under observation last year five were physicians. It is very possible that in the past years the rate was about the same,

Dr. Pettey of Memphis.

Replying to yours of recent date will say that 20 percent of the drug patients coming under my care for the past 12 months have been physicians and about that many more were members of physicians' families.

Dr. Allen of Milledgeville.

Aftergoing over all of my cases for the last 15 years, I find that I have had 134 inebriates, 18 doctors, 4 doctors' wives and 5 druggists, out of 744 patients. You will see from this that a very large per cent. of them are doctors in proportion to the number of all other classes compared with the doctors.

Dr. Burnett, who has a large sanatorium in Kansas City, in addition to answering my inquiry makes some statements which are so strirking that I reproduce the entire letter:

Yours at hand some days ago and will say that the winter number, 1906, of the Quarterly Journal of Inebriety pubished my last article on the subject, entitled "Pathology of the Morphine Habit and Treatment, "This article is based on 40 cases taken from my record just in order of admissions. The foot-note states: "The last 40 cases, with one exception, have either been physicians, members of physicians' families or nurses.

The explanation of this is that 30 were physicians, 6 were physician's wives; 3 were nurses; 1 was a druggist. The 6 wives were made morphine users by their husbands, who were physicians, and were addicted to the habit. These 40 cases were admitted during a period of 18 months, making a continuous record as they came and were unselected. I rarely see a case who is not a physician or his family associate, he usually making his wife or companion an addict that they may both enjoy the dissipation together.

The tendency of the physician addict to making himself companions in his debauchery, has been impressed upon me by several instances coming to my notice. One had his letter-heads announcing his special devotion to curing the habit; and in this way covered up his own acts and excused himself in the eyes of the public for being constantly in the company of numerous morphine users whom he generously supplied with morphine and whom he really taught to use the drug.

Those observations have shown me that the majority of physicians, addicted any great length of time, invariably surround themselves by morphine using companions either by hunting them up or by getting the desired persons into the habit primarily. One wife told me that her husband's morphine addiction was detected by her through his many surreptitious efforts to get her to take morphine. She accused him and he denied it, and finally one day he flew into a rage accused her of taking it and attempted to prove the accusation by forcibly taking hold of her and taking two tubes of morphine tablets from her coat pocket which he had quietly slipped in there while walking. He confessed, under drastic pressure, that he longed for his wife's companionship in the habit condition.

I could relate much along this line. The physicians and those associated in the handling of drugs, are the ones who make addicts of themselves or others or both."

I have never seen anything that would justify me in drawing the conclusions Doctor Burnett does, although I have frequently seen man and wife both addicted; more frequently with doctors, but also among the laity several times, and, I think there is always a tendency in any addict when seeing one suffering, to offer them morphine. This tendency makes the physician habitue particularly prone to the use of the drug in his practice.

As suggested in one of the letters, it is probable that doctors are more disposed to go to regular physicians and therefore the proportion estimated in these letters might be in excess of the actual condition, but on the othe rhand, they are very much disposed to keep their condition secret, of course, and on this account many do go to the advertizing institutions.

In my own work the percentage has been in the neighborhood of 40 per cent. and I should say that of all drug habitue sabout 25 per cent are physicians.

Considering the proportion of doctors to the general population, see how much greater the danger is for us.

The lecturers on therapeutics in the colleges cannot be too strong in cautioning medical students as to the dangers along this line, and should make a point to do so.

No physician should take a dose of morphine, of his own prescribing, except in a very extreme case, and particularly by the syringe, the danger of forming the habit in this way being greater.

In treating doctors for drug habits we have the same thing to contend with which makes it a common saying in hospitals, that doctors and nurses make the most troublesome patients, only intensified by their habits. Each one thinks he is different from others, and his case must be treated differently—remedies have a different and peculiar effect on him, etc.

